# Morphological and transcriptional analysis of *Colletotrichum lindemuthianum* race 7 during early stages of infection in common bean

**DOI:** 10.1590/1678-4685-GMB-2022-0263

**Published:** 2024-04-08

**Authors:** German Romero, Sandra González, Wendy Royero, Adriana González

**Affiliations:** 1 Universidad Nacional de Colombia, Facultad de Ciencias Agrarias, Bogotá, Colombia.; 2 Universidad Nacional de Colombia, Instituto de Biotecnología, Bogotá, Colombia.

**Keywords:** Anthracnose, RNAseq, CAZymes, Transporter protein, effector protein

## Abstract

The infection process of the hemibiotrophic fungus *Colletotrichum lindemuthianum* has been independently studied at the microscopic and genomic levels. However, the relationship between the morphological changes and the pathogenicity mechanisms of the fungus at the early stages of the infection remains uncharacterized. Therefore, this study attempts to bridge this gap by integrating microscopic and transcriptional approaches to understand the infection process of *C. lindemuthianum*. Fungal structures were followed by fluorescence microscopy for 120 hours. Simultaneously, the transcriptomic profile was made using RNAseq. Morphological characterization shows that appressoria, infective vesicles, and secondary hypha formation occur before 72 hours. Additionally, we assembled 38,206 transcripts with lengths between 201 and 3,548 bp. The secretome annotation revealed the expression of 1,204 CAZymes, of which 17 exhibited secretion domains and were identified as chitinases and β-1,3-glucanases, 27 were effector candidates, and 30 were transport proteins mostly associated with ABC-type. Finally, we confirmed the presence and expression of *CAC1* role during the appressoria formation of *Clr7*. This result represents the first report of adenylate cyclase expression evaluated under three different approaches. In conclusion, *C. lindemuthianum* colonizes the host through different infection structures complemented with the expression of multiple enzymes, where *CAC1* favors disease development.

## Introduction

The hemibiotrophic pathogen *Colletotrichum lindemuthianum* (Sacc. & Magn.) Bri. & Cava is the causal agent of anthracnose in common bean (*Phaseolus vulgaris* L), one of the most important fungal diseases worldwide. Bean anthracnose could cause production losses up to 100 % depending on the host susceptibility and the existence of favorable climatic conditions for the pathogen growth (Campa et al., 2014; Padder et al., 2016).

The symptoms of anthracnose are characterized by dark sunken lesions with the presence of salmon-colored acervuli. Necrosis has been reported in petioles, pods, and leaves, accompanied by strangulation of the hypocotyl and seed discoloration (Melotto et al., 2000; Schwartz et al., 2005; Dean et al., 2012). Many genotypes of beans express these symptoms in different grades due to the high genetic variability of *C. lindemuthianum* (Dean *et al.*, 2012). To date, there are more than 200 races of this pathogen reported worldwide (Pinto et al., 2012; Martiniano-Souza et al., 2017). In Colombia, bean-producing areas present a high incidence of races 7, 13, 133, 137, 139, 521, and 645 (Gallego et al., 2010). Race 7, hereafter *Clr7*, is highly virulent in different established bean cultivars in the country (Pedroza et al., 2022).

*C. lindemuthianum* lifestyle has been studied at the morphological level using microscopy techniques such as transmission electron (O’Connell et al., 1985) and fluorescence microscopy (O’Connell and Bailey, 1991; Perfect et al., 1999). These studies enabled the characterization of the hemibiotrophic lifestyle, outlining two stages in the infection process. The first is the biotrophic phase, which involves the formation of appressoria and peg penetration during the first 24 hours and the formation of the infective vesicle between 48 and 72 hours (Rawlings et al., 2007). The second stage corresponds to the necrotrophic phase, which includes the transformation of primary hyphae to secondary hyphae at inter and intracellular levels between 72 and 96 hours after penetration (O’Connell *et al.*, 1985). However, there is no report of a morphological characterization of the infection process of *C. lindemuthianum* in prevalent common beans races in Colombia.

High Throughput Sequencing (HTS) is a powerful alternative for the analysis of gene expression. The large-scale sequencing of messenger RNA (RNAseq) has achieved greater sensitivity and coverage of the entire transcriptome to an unprecedented level (Trapnell et al., 2010). The RNAseq methodology applied to eukaryotic pathosystems (e.g., *Colletotrichum higginsianum* - *Arabidopsis thaliana*, and *Colletotrichum graminicola* - *Zea mays*) has allowed for the identification of putative effectors, secondary metabolites, and other genes whose expression are determinant in the infection processes (O’Connell et al., 2012). The transcriptomic studies in the pathosystem *C. lindemuthianum - P. vulgaris,* have been focused on the host molecular response, particularly on the detection of differentially expressed genes encoding resistance proteins in the host (Padder et al., 2016; Alvarez-Diaz et al., 2022). However, from the pathogen´s perspective, only one study has described a repertoire of effector candidate genes, and no study has performed a transcriptomic characterization of *Clr7* at different times post-infection during an *in vivo* assay (Padder *et al.*, 2016; de Queiroz et al., 2019; Alvarez-Diaz *et al.*, 2022). Therefore, we characterized for the first time the infection process of *Clr7* at morphological and transcriptomic levels, establishing the correlation between the infection structures and gene repertoire from the pathogen *in planta*.

## Material and Methods

### Plant material

Bean seeds of the Sutagao cultivar were sown in propagation trays containing 50 alveoli with peat. The plants were grown for 5 days under semi-controlled greenhouse conditions at 18 °C, 70 % relative humidity, and 12 h light /12 h dark photoperiod.

### Inoculation of *Colletotrichum lindemuthianum* race 7

Race 7 of *C. lindemuthianum* (*Clr7*) was supplied by the Plant Health Laboratory of the Faculty of Agricultural Sciences at the Universidad Nacional de Colombia. To produce the fungal biomass, the fungus was cultivated in sterile bean pods at 17 °C for 15 days, following the protocol proposed by the International Center for Tropical Agriculture (CIAT) (Castellanos et al., 2011). The produced inoculum was adjusted to a concentration of 1X10^7^ conidia mL^-1^. Both sides of the first pair of true leaves, at seven days after sowing, were entirely brushed with *Clr7* inoculum. The control treatment consisted of simulating the inoculation process in same-age plants with sterile distilled water. To ensure pathogen infection, plants were covered with plastic bags for 24 h in order to maintain relative humidity above 90% and were kept under greenhouse conditions at 18 °C and 12 h light /12 h dark photoperiod.

### *In vivo* characterization of the infection process in *Colletotrichum lindemuthianum* race 7

The leaves of two plants were randomly harvested at 0, 24, 48, 72, 96, and 120 hours after inoculation (hai), and then individually immersed in a 1 M KOH solution with a drop of Tween 80 for 24 h. Each leaf was subsequently immunofluorescent stained with wheat germ agglutinin - Fluorescein isothiocyanate (WGA-FITC) at a concentration of 20 μg mL^-1^ (L4895-10MG; Sigma-Aldrich) according to the protocol proposed by Dawson et al. (2015).

Fungal infective structures were visualized at the morphological level using a fluorescence microscope with a GFP filter (Nikon Eclipse TI) at 40x. Measurements were taken from 10 cuts of 1 cm^2^ for each of the evaluated times. The considered measurements were conidia length and diameter, appressoria diameter, infective hyphae length, the length between septa and diameter of primary hyphae, and length between septa and diameter of secondary hyphae. All measurements were made with the program ImageJ (ver. 1.8.0_172).

### RNA extraction, library preparation, and sequencing

The first two true leaves, previously inoculated with the suspension of conidia or water, were harvested at 0, 24, 48, and 72 hai. Once removed, they were frozen in liquid nitrogen and stored at -80 °C. RNA extraction was performed following the CTAB-based protocol methodology with some modifications made by Pedroza et al. (2022). Total RNA was treated with DNase I (RNase-free DNase Set, Qiagen). The absence of DNA was verified through the amplification of the actin gene (α-Actin) of *P. vulgaris*. PCR reaction was performed in a volume of 15 µL composed of 1 X Buffer, 2.5 mM MgCl_2_, 0.25 dNTPs, 0.1 µM of each primer, and 1 U of *Taq* polymerase. The reaction was performed under the following thermal profile: 95 °C for 3 min, followed by 34 cycles of 95 °C for 30 s, 61 °C for 40 s, 72 °C for 2 min, and finally an extension for 72 °C for 5 min.

RNA quality, in terms of integrity and concentration, was evaluated by agarose gel electrophoresis and measured with RIN (RNA Integrity Number) and NanoDrop™ One, respectively. The samples that accomplished the quality requirements were sequenced by Illumina’s technology (San Diego, CA) by Novogene (Beijing, China). For that, RNA libraries were built using the TruSeq cDNA kit and the sequencing was performed using the NovaSeq™ 6000 instrument, paired-end 150 bp (PE150).

## Preprocessing and computational analysis

### Preprocessing

Raw data quality was evaluated through FastQC v. 0.11.8 (Wingett and Andrews, 2018). Adapters and low-quality reads were removed with Trimmomatic (ver. 0.38) using a minimum threshold of Q20 (Parameters: sliding window: 4:20 trailing: 3 minlen: 130) (Bolger et al., 2014).

### Sequence filtering

To remove host and microbial-associated sequences belonging to the interaction and keep only the sequences from *Clr7* for the transcript assembly process, we carefully selected the sequences belonging to the Fungi kingdom. For this, the pre-processed readings were taxonomically classified to identify their origin by homology using the program Kaiju (v. 1.7.2) (Menzel and Krogh, 2016). Sequences belonging to other domains, such as bacteria and plants, were identified and then excluded. Subsequently, the genomes of the contaminant species were downloaded and used as input for the subtraction through a mapping process with the BBduk tool (v. 35.38). The mapped sequences were separated into independent files for further analysis. Finally, we ensured the absence of contaminant reads by performing a second round of filtering with the ContFree-NGS program (Peres and Riaño-Pachón, 2021).

### Assembly of the transcriptome

The fungal transcriptome was assembled independently for each of the three evaluated times (24, 48, and 72 hai) using a *de novo* approach with Trinity software (v. 2.8.5) (Haas et al., 2013). Default parameters were used, and sequences with low *k-mer* coverage were discarded.

### Functional Annotation

To determine putative functions and detect homology between the predicted open reading frames (ORFs) with potential proteins, we adopted the following pipeline. ORFs were detected using TransDecoder (ver. 5.5.0). After ORFs were extracted, we performed a general functional annotation using the program EggNog (Evolutionary genealogy of genes: Non-supervised Orthologous Groups) (ver. 4.5.1). In this way, clusters of orthologous groups (COG categories) and gene ontology (GO) were obtained based on their similarity with the European Bioinformatics Institute database (EBI) and The Uniprot Consortium database (Bateman et al., 2020).

### Secretome annotation

Pathogenicity-related genes were annotated identifying functions such as Carbohydrate-Active enzymes (CAZymes), membrane transporters, and Candidates to Secreted Effector Proteins (CSEPs). Secreted and non-secreted CAZymes present in the transcript-assembled catalog were identified by searching for conserved unique peptide patterns (CUPP) using the CUPP platform (Barrett et al. 2020). 

Membrane transport proteins were tracked by homology mapping of the total number of transcripts against the Transporter Classification Database (TCDB) (Saier et al., 2021) through the Blast tool (ver. 2.8.1) using an e-value of 1e-25. For CSEP annotation, we identified the presence of a signal peptide in the transcript catalog using SignalP (ver. 5.0) (Almagro et al., 2019). Then, we followed the pipeline reported by de Queiroz et al. (2019). Proteins that exhibited a length of fewer than 300 amino acids were used as input to the effector function prediction using the matching learning tool EffectorP (ver. 3.0) (Sperschneider and Dodds, 2021). Finally, putative effector catalog functions were annotated by mapping them against the Pathogen Host Interaction database using an e-value of 1e-3 (Urban et al., 2019).

### Validation of Adenylate cyclase gene *CAC1* expression *in planta*

Specific primers were designed with the Primer-Blast designing tool from NCBI and synthesized to amplify *CAC1,* the *mac1* homolog gene detected in the transcriptomic analysis (Table 1). *C. lindemuthianum* race 7 DNA was used to verify the amplification of *CAC1*, which codes for the adenylate cyclase gene, detected at 48 hai by transcriptomic analysis in this work. The ITS 1-4 region was used as an amplification control (Table S1). Each PCR reaction contained 1X PCR Buffer, 2.5 mM MgCl_2_, 0.25 mM of each dNTP, 0.25 µM of each primer, 0.5 U of Taq polymerase (Thermo), and 2 µl of DNA template. The PCR reaction mix was adjusted to a final volume of 20 µl with nuclease-free water. The PCR amplification program consisted of an initial step of 2 min at 95 °C, followed by 30 cycles of 1 min at 95 °C, 30 s at 60 °C, and 2 min at 72 °C; and a final extension step of 10 min at 72 °C. PCR products were analyzed in 3% agarose gel, stained with SYBR safe, and visualized under UV light. The Low Molecular Weight DNA Ladder (New England BioLabs) was used as a molecular weight marker.

**Table 1:** Functional annotation of candidate secreted effector proteins (CSEPs) identified in the infection process of *Colletotrichum lindemuthianum* race 7 and their homologous reported in other fungal pathogens.

Time (Hours)	Gene name	% ID	e-value	Protein annotation	Uniprot Accesion	Reported fungal pathogen	References
24	*CBP1*	64,51	1.16e-05	Chitin deacetylase	G4N4F9	*Magnaporthe oryzae*	Dean et al., 2005
24	*bcsod1*	56,20	9.82e-54	Superoxide dismutase	Q70Q35	*Botrytis cinerea*	Rolke et al., 2004
24	*CBP1*	80,00	5.06e-05	Chitin deacetylase	G4N4F9	*Magnaporthe oryzae*	Dean et al., 2005
24	*MoPtc2*	43,13	6.53e-06	Protein phosphatase 2C	Q4WTH5	*Magnaporthe oryzae*	Nierman et al., 2005
24	*CBP1*	81,81	3.10e-06	Chitin deacetylase	G4N4F9	*Magnaporthe oryzae*	Dean et al., 2005
24	*MGG_10510*	56,61	4.53e-10	Ribonuclease T2	G4MS40	*Magnaporthe oryzae*	Dean et al., 2005
24	*mac1*	33,33	7.50e-12	Adenylate cyclase	O13328	*Magnaporthe oryzae*	Dean et al., 2005
48	*bcsod1*	55,92	3.54e-50	Superoxide dismutase	Q70Q35	*Botrytis cinerea*	Rolke et al., 2004
48	*FZC9*	33,71	1.37e-26	Transcriptional activator xlnR	G5EH28	*Magnaporthe oryzae*	Dean et al., 2005
48	*CBP1*	64,52	1.27e-05	Chitin deacetylase	G4N4F9	*Magnaporthe oryzae*	Dean et al., 2005
48	*CRU1*	38,48	7.43e-06	Cell cycle regulatory protein	Q8J214	*Ustilago maydis*	Unpublished
48	*mac1*	40,23	1.42e-09	Adenylate cyclase	O13328	*Magnaporthe oryzae*	Choi and Dean, 1997
48	*CBP1*	81,82	2.70e-06	Chitin deacetylase	G4N4F9	*Magnaporthe oryzae*	Dean et al., 2005
48	*PDIG_23520*	57,34	4.72e-47	Cysteine-rich Anionic (Sca) protein	K9G4Z7	*Penicillium digitatum*	Marcet-Houben et al., 2012
48	*CBP1*	80,00	1.15e-04	Chitin deacetylase	G4N4F9	*Magnaporthe oryzae*	Dean et al., 2005
48	*GzGPB1*	53,02	1.19e-83	G protein beta subunit	I1RJS2	*Fusarium graminearum*	Cuomo et al., 2007
48	*Pstg_13661*	45,30	1.01e-47	chitin deacetylase	A0A0L0V0Y3	*Puccinia striiformis*	Duplessis et al., 2011
48	*UvHrip1*	69,52	5.73e-53	Effector protein	A0A1B5KZM5	*Ustilaginoidea virens*	Kumagai et al., 2016
72	*CBP1*	81,82	2.34e-06	Chitin deacetylase	G4N4F9	*Magnaporthe oryzae*	Dean et al., 2005
72	*CBP1*	41,07	3.99e-05	Chitin deacetylase	G4N4F9	*Magnaporthe oryzae*	Dean et al., 2005
72	*Ubc9*	38,00	9.09e-05	SUMO-conjugating enzyme ubc9	G4ND54	*Magnaporthe oryzae*	Dean et al., 2005
72	*DCL1*	39,01	5.07e-24	Dicer-like protein 1	A0A194VW85	*Valsa mali*	Yin et al., 2015
72	*Mouba2*	51,13	1.44e-93	Ubiquitin-activating enzyme E1-like	G4MLK2	*Magnaporthe oryzae*	Dean et al., 2005
72	*Pstg_13661*	47,74	1.11e-45	chitin deacetylase	A0A0L0V0Y3	*Puccinia striiformis*	Duplessis et al., 2011
72	*LmABCB3*	36,13	2.69e-17	MDR efflux pump ABC3	Q3Y5V5	*Magnaporthe oryzae*	Dean et al., 2005
72	*bcsod1*	56,21	9.82e-54	Superoxide dismutase	Q70Q35	*Botrytis cinerea*	Rolke et al., 2004
72	*MGG_10510*	26,71	3.44e-10	Ribonuclease T2	G4MS40	*Magnaporthe oryzae*	Dean et al., 2005

The primer specificity was confirmed by sequencing of PCR product using the Sanger method. The DNA template was quantified by Fluorimeter using QubitTM dsDNA HS Kit (Invitrogen®). A 10-fold dilution series (1 to 1/10.000.000) was used for the standard curve and melting curve analysis which were run in qTOWER 3 Real-Time PCR Thermal Cycler. This program consisted of an initial step of 2 min at 95 °C, followed by 30 cycles of 1 min at 95 °C, 30 s at 60 °C, 2 min at 72 °C; and a final extension of 10 min at 72° C. This was followed by a final melting step. The PCR reaction mix contained 1X BlasTaq™, 2X qPCR MasterMix (Applied Biological Materials), 0.12 µM of each primer, 1 µl of DNA, and it was adjusted to a final volume of 10 µl. The efficiency (E) of the primer was calculated using a linear regression model according to the equation: E = (10^[− 1/slope]^ − 1) × 100 (Pfaffl, 2001).

The validation of *CAC1* expression was performed with a pool of RNA of three leaves of the Sutagao cultivar inoculated with *Clr7* from different post-inoculation times (24, 48, and 72 hpi). For this, one-step reverse transcription-polymerase chain reaction (qRT-PCR) was performed in a reaction containing 1X Master Mix (Thermo Fisher), 0.12 µM of each primer, and 3 µl of RNA. The real-time qPCR Thermal Cycler program consisted of one initial step of 2 min at 95 °C, followed by 40 cycles of 1 min at 95 °C, 30 s at 51 °C, 2 min at 72°C; and a final extension step of 10 min at 72 °C. The melting curve was continuously generated under the profile at 95 °C for 5 s, 60 °C for 1 min, and 95 °C. For the relative quantification, the Cp (crossing point) values of the *ClrRNA2* housekeeping gene and *CAC1* gene were compared, considering the amplification efficiency of each gene. Three technical replicates were included for each gene and evaluation time. The expression levels were determined using the Pfaffl method (Pfaffl, 2001), and the log2-transformed fold change of *CAC1* was calculated.

## Results

### Pivotal morphological changes of *Colletotrichum lindemuthianum* race 7 occurred before the first 72 hours of the host infection

**Figure 1-:**
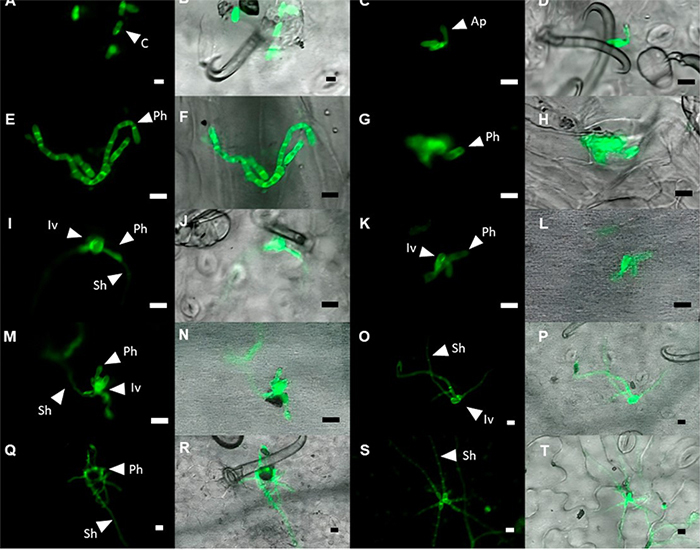
Follow-up of the *in planta* infection process of *Colletotrichum lindemuthianum* race 7 in the bean cultivar Sutagao at 24 hai (A - D), 48 hai (E - H), 72 hai (I - L), 96 hai (M - P) and 120 hai (Q - T). The morphological characterization was performed on the following structures: C: Conidia, Ap: Appressoria, Iv: Infective vesicle, Ph: Primary hypha, and Sh: Secondary hypha. Black and white bars: 10 µm.

The infection process of *Clr7* started 24 hai with the penetration processes of the fungus. Figures 1 A and C show cylindrical and not septate conidia, typical of this fungus. The measures taken at 24 hai on histological sections of *C. lindemuthianum* (*Clr7*) and *P. vulgaris* interaction showed that conidia average length and average diameter were 14.29 µm (+ 1.4 µm) and 4.92 µm (+ 0.47 µm), respectively. During this time series, the formation of both the germinal tube and the appressoria was observed, with the latter exhibiting an average diameter of 5.48 µm. 

Microscopic analysis showed that the biotrophic phase of *Clr7* began at 48 hai and was characterized by the presence of infective hyphae with a circular and irregular shape. The average of the infective hypha was 8.87 µm (+ 1.77 µm) (Figure 1 G ). Additionally, the formation of the primary hyphae was observed, with an average septa length of 9.23 µm (+ 1.3 µm) and an average diameter of 6.95 µm (+ 0.5 µm) in the biotrophic base (Figure 1 E-H ).

The main transitional change between biotrophic and necrotrophic phases was observed at 72 hai and constituted the transformation of primary hypha into secondary hypha (Figure 1 I-L ). The secondary hypha of *C. lindemuthianum* (*Clr7*) exhibited cells with a smaller diameter and greater length than primary hypha -2.25 µm (+ 0.44 µm) and 14.77 µm (+ 1.71 µm), respectively. Finally, the histological sections at 96 and 120 hai demonstrated secondary hypha extension and adjacent cell colonization (Figure 1 M-T ).

### Repertoire-expressed genes of the infection process of *Colletotrichum lindemuthianum* race 7 were revealed by transcriptomic analysis

The RNA sequencing of the different time series of *C. lindemuthianum* and *P. vulgaris* interaction generated between 39.9 and 53 million reads, more than 92.7 % accomplished the Q20 and Q30 quality parameters and contained an indeterminate number of bases lower than 0.01 % according to FastQC results (Table S2). The guanine-cytosine average content of reads was 45.8 %, and less than 3.8 % of these datasets corresponded to adapter sequences. The replica sequences from sample times 24, 48, and 72 hai were concatenated, the adapters and rRNA were removed, and a total of 37.2 to 42.6 million reads were obtained. 

The taxonomic composition analysis revealed that 50.5 % of the reads were related to the Bacteria domain with relevance to the phylum Proteobacteria (*Acinetobacter* sp., *Pseudomonas* sp., and *E. coli* sp.), 11 % to the Fungi kingdom (underlining species of phylum Ascomycota), and 22 % to the clade Viridiplantae. We confirmed that 99.9 % of the filtered reads reliably belonged to the Fungi kingdom. The filtering process left a total of 1,660,766 and 2,277,685 reads, representing 4.78 % of the total sequences obtained in the infection process (Table S3).

### Ortholog clusters related to the growth and development of *C. lindemuthianum* race 7 were revealed by computational analysis

The *Clr7* transcriptome profile comprised a total of 38,206 transcripts, with 13,211 obtained at 24 hai, 12,962 at 48 hai, and 12,033 at 72 hai. The assembled transcripts presented a mean length of 542 bp, ranging from the shortest at 201 bp to the longest at 3,548 bp. GO assignment results revealed that 61 % (n=38,206) of the obtained transcripts were associated with biological processes, 38 % (n=38,206) with molecular function, and 1 % (n=38,206) with cellular components. Additionally, we detected different protein family domains (PFAM), where the main PFAM categories involved in the infection process corresponded to unknown function (3,205 transcripts), post-translational modifications (2,954), transduction (2,826), translation (2,586), and RNA modification (1,904) (Figure S1).

### CAZymes secreted repertoire was revealed in the infection process of *Colletotrichum lindemuthianum* race 7

**Figure 2 -:**
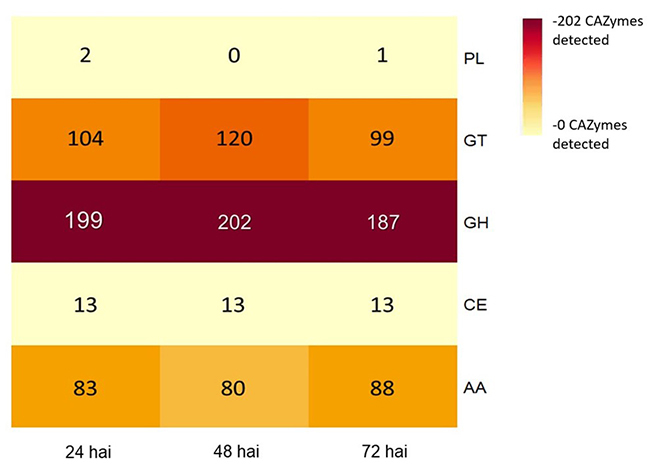
Heat map of the number of CAZymes detected with CUPP platform during 24, 48, and 72 hai of the race 7 of *Colletotrichum lindemuthianum* infection process. Glycosyl hydrolases (GH), Glycosyl transferases (GT), Enzymes with auxiliary activity (AA), Carbohydrate esterases (CE), and Polysaccharide lyases (PL).

A total of 1,204 CAZymes were identified in the early stages of infection, with 401, 415, and 388 transcripts related to CAZymes annotated at 24, 48, and 72 hai, respectively. Four classes of CAZymes were detected with no difference between times of evaluation. Glycosyl hydrolases (GH) were the most strongly represented with 48.8 % (n=1,204), followed by glycosyl transferases (GT) 26.8 % (n=1,204), enzymes with auxiliary activity (AA) 20.8 % (n=1,204), carbohydrate esterases (CE) 3.2% (n=1,204), and finally, the group of polysaccharide-lyases (PL) at 0.2 % (n=1,204) (Figure 2). The CAZymes composition analysis annotated a total of 72 families, with 31 identified as GH, highlighting GH152, GH17, GH31, and GH35; 29 as GT, highlighting GT1, GT4, and GT22; nine as AA, underlining AA1, AA3, and AA6; four as CE (CE8, CE14, CE4, and CE5), and two associated with PL (PL1 and PL3). Finally, a total of 17 CAZymes with extracellular activity were detected in the infection process with a dominance of the GH152 family (enzymes with β-1,3-glucanase activity), followed by the GH18 and GH19 family (chitinases) (Table 2).

**Table 2 -:** CAZymes with secretion domains identified through the CUPP database in the early stages of the infection process of *Colletotrichum lindemuthianum* race 7.

Hours after inoculation (hai)	CAZyme Family	Total	Enzyme description
24	GH3	2	Xylan 1,4-β-xylosidase
24	GH18	1	Chitinase/lysozyme
24	GH19	4	Chitinase
24	GH152	9	β-1,3-glucanase
48	CE4	1	Chitin deacetylase
48	GH3	1	Xylan 1,4-β-xylosidase
48	GH16	1	Xyloglucan:xyloglucosyltransferase
48	GH18	1	Chitinase/lysozyme
48	GH19	4	Chitinase
48	GH152	7	β-1,3-glucanase
72	CE4	1	Chitin deacetylase
72	GH1	1	β-glucosidase
72	GH3	1	Xylan 1,4-β-xylosidase
72	GH18	1	Chitinase/lysozyme
72	GH19	1	Chitinase
72	GH27	1	α-galactosidase
72	GH152	7	β-1,3-glucanase

### ABC-type are the main transport proteins involved in the infection process of *Colletotrichum lindemuthianum* race 7

A total of 30 transporter proteins were identified in the infection process - 12 at 24 hai, eight at 48, and 10 at 72 hai. Transporter protein ABC-type was the most abundant in the datasets with 23.3 % (n=30), followed by α-mannosidase I with 13.3 % (n=30). Moreover, proteins associated with sugar and phospholipid transport were identified at 48 and 72 hai (Figure S2).

### A repertoire of candidate genes for secreted effector proteins was detected and shows similarity with effectors of known plant pathogens

**Figure 3 -:**
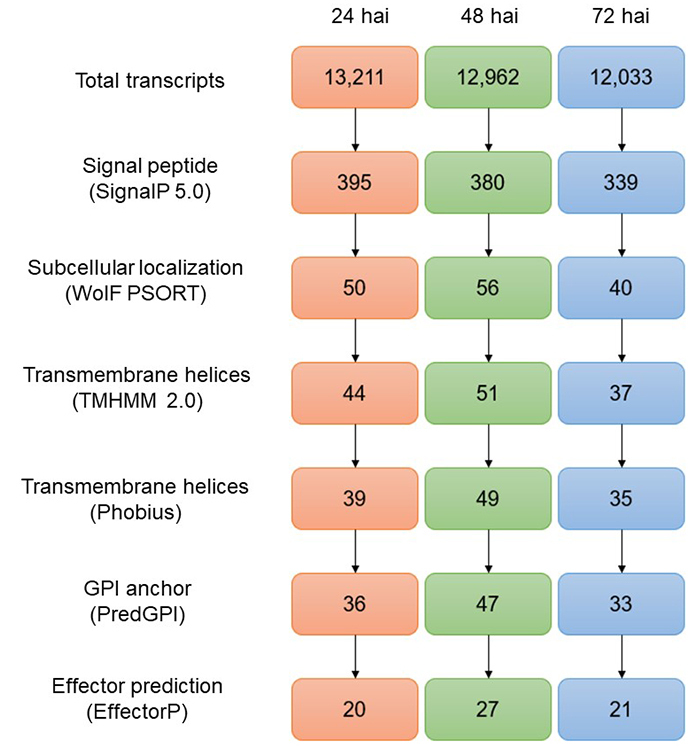
Candidates for secreted effector proteins (CSEPs) obtained during the infection process of *Colletotrichum lindemuthianum* at 24, 48, and 72 hours. The number inside the boxes represents the sequences that passed each of the pipeline filtering steps.

A total of 1,114 sequences with peptide signals were annotated, of which 6.1 % (n=68) were identified as CSEPs (Figure 3). The location prediction of effector candidates revealed that 85.2 % might have apoplastic activity, while 7.4 % may have cytoplasmic activity. Twenty-seven CSEPs presented homology with effector proteins reported in the PHI database. Of these, seven, 11, and nine were annotated at 24, 48, and 72 hai, respectively (Table 1). Additionally, these homolog effectors were distributed across 14 PFAMs related to chitinases at 29.6 % (n=14), superoxide dismutase at 11.1 % (n=14), proteins with leucine-rich repeats (LRR) kinase activity at 7.4 % (n=14), and ribonuclease at 7.4 % (n=14).

### *CAC1* is a candidate-secreted effector protein at the early stages of infection

**Figure 4 -:**
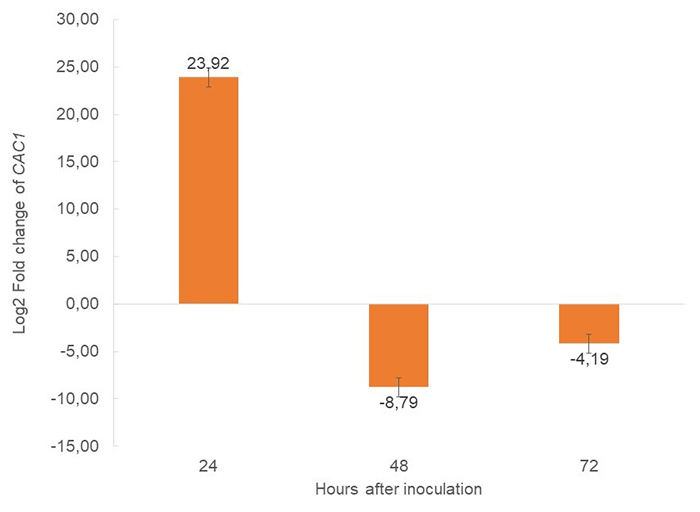
Relative expression of *CAC1* by quantitative real time PCR (qRT-PCR). X-axis represents hours after inoculation of *C. lindemuthianum* race 7 in Sutagao cultivar; Y-axis represents the log of the relative quantitation value. Errors bars are the standard errors from three technical replicates. The expression levels were determined using the Pfaffl method, the values corresponds to the log2-transformed fold change of *CAC1*.

The transcriptomic analyses showed a repertoire of candidate genes involved in the infectious process of *Clr7*. From these, *CAC1*, an adenylate cyclase coding gene, was selected to validate its expression at 24, 48, and 72 hai. The relative expression analysis showed a higher expression of *CAC1* at 24 hai compared with the other infection times, clearly demonstrating repression in the expression levels (Figure 4).

## Discussion

The infection process of *Clr7* in the bean cultivar Sutagao was classified into three stages throughout the 120 hours of evaluation: pre-penetration, infection of the pathogen (biotrophic phase), and colonization (necrotrophic phase). In the first stage, the events of conidia adhesion, tube formation, and appressoria development were detected by fluorescence microscopy at 24 hai. The pathogen structures identified at 24 hai corresponded with *in planta* and *in vitro* findings made previously by electron and fluorescence microscopy (O’Connell et al., 1985; Herbert et al., 2004). The diameter of the conidia ranged between 10.5 and 14.5 µm, maintaining a cylindrical shape consistent with the characteristics reported for this fungal species. Likewise, the dome-type appressoria had a spherical shape with an average diameter that fits, as reported by Liu et al. (2013). The pre-penetration and penetration processes in *C. lindemuthianum* are essential for disease establishment. Following conidial adhesion, germination occurs, leading to the production of melanized appressoria. This structural feature not only protects itself from environmental radiation but also creates favorable conditions for host penetration through the peg, facilitated by the pressure exerted by the appressoria (Saint et al., 2015). Consistent with O’Connell and Bailey (1991), we observed the biotrophic phase at 48 hai, characterized by the formation of infective vesicles and primary hyphae. Although the infective vesicles exhibited a slightly larger average diameter compared to the findings of  O’Connell *et al.* (1985), with a difference of 0.87 µm, the formation of these structures ensured the successful establishment of the pathogen within the host (O’Connell *et al.*, 2012).

Similar to other reports, the necrotrophic phase of *C. lindemuthianum* was observed at 72 hai. However, in *Colletotrichum truncatum* the start of necrotrophy has been described between 56 and 68 hours (Bhadauria et al., 2011; 2013). This phase was characterized by the differentiation of primary hypha into secondary hypha; the latter showed a diameter within the range reported for *C. lindemuthianum* (O’Connell and Bailey, 1991). The emergence of secondary hyphae from the primary hypha marked a crucial transition associated with the disruption of the host protoplast and the subsequent colonization of adjacent cells, as documented by O’Connell *et al.* (1985). For this process, the pathogen requires the secretion of virulence factors, such as enzymes responsible for removing pectin. This could be associated with the PL1 family pectate-lyases detected in the *in silico* analysis at 72 hai.

The transcriptional characterization revealed that the main category associated with the infection process of *C. lindemuthianum* corresponded to proteins related to post-translational modifications, protein translation, RNA processing, and carbohydrate metabolism. This is related to the cell cycle of the pathogen, involving growth and development functions (Cantarel et al., 2009). Additionally, proteins related to post-translational modifications, such as certain heat shock proteins (HSPs) were detected. These proteins can be expressed under conditions of biotic or abiotic stress and function as modulators of virulence, as seen with the chaperone HSP90 in *Candida* sp. (O’Meara et al., 2017).

A wide variety of CAZymes was found at all the evaluation times. CAZymes are key in the infection process of *C. lindemuthianum* because they degrade the host’s cell wall and modify the cell wall of the fungus (O’Connell et al., 2012; da Costa et al., 2021). Glycosyl hydrolases (GH) constitute a class of CAZymes with the activity to hydrolyze carbohydrates, proteins, or lipids. Within this class, the GH5, GH16, GH18, GH13, GH31, and GH61 families have been reported to participate in the infection process of phytopathogen fungi (Zhao et al., 2013; Liang et al., 2018). This is consistent with our results that showed a dominance of the GH152, GH31, GH3, GH17, and GH16 families.

CAZymes with secretion domains were expressed at 24, 48, and 72 hai and those with β-1,3-glucanase enzymatic activity (GH152) were dominant. These enzymes play a crucial role in the modification processes and reorganization of the fungal cell wall architecture during the growth and cell differentiation of the pathogen. It has been evidenced in *Aspergillus fumigatus* (Mouyna et al., 2013) and *Magnaporthe oryzae* (Wang et al., 2021).

The detection of chitinases from GH18 and GH19 families further supports their significant role in the infection process of *C. lindemuthianum*. Chitinases play a crucial role in modifying the fungal cell wall and promoting the growth and development of the pathogen (Fiorin et al., 2018). Additionally, chitinases can suppress PTI-type immunity by hydrolyzing chitin, thus preventing its recognition by plant pattern-recognition receptors (PRRs). This modulation of the host immune response is a common strategy employed by pathogens to facilitate successful infection, as exemplified by MoChi1, a chitinase in *M. oryzae*, and OsMBL1, a PRR in rice (Han et al., 2019). 

During the infection process of *C. lindemuthianum*, it was possible to detect ABC-type transport proteins more frequently in all evaluation times. These proteins are involved in the transport of different compounds through the cell membrane such as xenobiotics and metabolites, which are directly involved in the pathogenesis of several species of filamentous fungi (Schoonbeek et al., 2002; Víglaš and Olejníková, 2021). In fungal species such as *M. oryzae* and *Colletotrichum acutatum*, mutants with a deficiency in the ABC transporter protein are more susceptible to antifungal compounds such as phytoalexins, reducing the establishment of the fungus in cytotoxic environments (Sun et al., 2006; Gupta and Chattoo, 2008; Kim et al., 2014).

The mechanisms of pathogenesis in *C. lindemuthianum* are regulated in the biotrophic phase (the stage during which the emission of the effectors occurs). These molecules are the main pathogenicity determinants that suppress the plant defenses by gene silencing or signaling cascade disruption (de Queiroz et al., 2019). The *In-silico* analysis revealed a total of 27 potential candidates secreted effector proteins (CSEPs). At 24 hai, the presence of adenylate cyclase homolog protein was detected, which is an enzyme crucial for penetration into the host and the formation of the appressoria (Choi and Dean, 1997).

At 24 and 48 hai, a protein homologous to the mitogen-activated protein kinase (MAPK) protein PMK1 was detected. It is involved in the formation of the appressoria and the infective hypha growth in *M. oryzae* (Bruno et al., 2004; Bartels et al., 2009). Likewise, this protein regulates key functions in the host invasion process, such as cytoskeleton reorganization, exocytosis, and expression of effector genes that are key in this phase (Osés et al., 2021). At this early stage of infection, we also detected and validated the expression of a CSEP (*CAC1*) that codifies an adenylate cyclase. The activity of *CAC1* is considered essential for successful fungal infection due to its role in MAPK cascades and cyclic AMP (cAMP) signaling pathways (Yamauchi et al., 2004; Fu et al., 2022). *CAC1* protein activity is described as crucial for the colonization process of several pathogens (e.g., *Magnaporthe oryzae, Fusarium graminearum, Colletotrichum lagenarium*, and *Colletotrichum scovillei*). *CAC1* mutant strains are less pathogenic and exhibit defects in conidial germination and appressoria formation (Yamauchi *et al.*, 2004; Zhou et al., 2012; Bormann et al., 2014; Yin et al., 2018; Fu *et al.*, 2022). As a part of this work, we confirmed the expression of *CAC1* in the early stages of *Clr7* infection using both transcriptomic and RT-qPCR approaches. The *CAC1* expression was detected at 24 and 48 hai by the transcriptomic strategy, as its homolog *mac1* (Table 1). Likewise, we confirmed the expression of *CAC1* at 24 hai in the validation assay by RT-qPCR (Figure 4). The CAC1 detected expression is compatible with the appressoria formation process also confirmed by microscopy in the morphological characterization. Together, these results underscore the crucial role of the *CAC1* in the infection process of *Clr7* and represent the first report of adenylate cyclase expression in this pathosystem evaluated using three different approaches. Eleven possible effector proteins related to the biotrophic phase of *C. lindemuthianum* were identified at 48 hai, one being the protein homologous to UvHrip1. This effector protein, detected in *Ustilaginoidea virens* is capable of suppressing the host defenses, programmed cell death, and ROS accumulation in *Nicotiana benthamiana* (Li *et al.*, 2020).

Finally, at the beginning of the necrotrophic phase, corresponding to 72 hai, a superoxide dismutase was detected. This enzyme plays a key in the pathogenesis of necrotrophic species, such as *Botrytis cinerea*, by modulating the production of H_2_O_2_ by induction of the hypersensitivity response (Azevedo et al., 2008; López et al., 2016). We also detected proteins related to SUMO-type post-translational modifications, such as homologous proteins to Ubc9 and MoUBA2 of *M. oryzae*. The last one is a determinant of pathogenesis, involved in the different infection processes such as mycelium growth, septa formation, and appressoria formation (Lim et al., 2018). 

In conclusion, during the first 72 hours after inoculation, *Clr7* colonizes the host through different infection structures. This colonization is complemented by the expression of multiple enzymes, such as chitinases, and β-1,3-glucanases, as well as regulator proteins like *CAC1*, which is essential for successful fungal infection due to its role in MAPK cascades and cyclic AMP (cAMP) signaling pathways. Additionally, there is the expression of effector proteins like homologous UvHrip1, along with superoxide dismutase-type and Sumo-type proteins that favor the establishment of the pathogen and the development of the disease.

## Supplementary material

The following online material is available for this article:

Table S1 - Primer sequence to validate gene expression of predicted candidate-secreted effector proteins.

Table S2 - Quantity and quality of reads from 0, 24, 48, and 72 hours after inoculation. Control (C) and inoculated (I) samples are presented.

Table S3 - Number of total reads concatenated for *Colletotrichum lindemuthianum* race 7, obtained after preprocessing and filtering for fungi kingdom.

Figure S1 - COG categories of *Colletotrichum lindemuthianum* race 7 identified through alignments between the EggNOG database and amino acid sequences obtained from *de novo* assembly.

Figure S2 - Transport proteins of *Colletotrichum lindemuthianum* race 7 identified in the Transporter Classification Database.
