# Supplementary material for: Morphological and transcriptional analysis of Colletotrichum lindemuthianum race 7 during early stages of infection in common bean
Source: Genet Mol Biol. 2024 Apr 8;47(1):e20220263. doi: 10.1590/1678-4685-GMB-2022-0263 (PMC11003654; doi:10.1590/1678-4685-GMB-2022-0263)
Supplement: Table S1 - [file 1415-4757-GMB-47-01-e20220263-s1.pdf]

Genetics and Molecular Biology

https://doi.org/10.1590/1678-4685-GMB-2022-0263

Supplementary Material to Morphological and transcriptional analysis of *Colletotrichum lindemuthianum* race 7 during early stages of infection in common bean

Table S1 - Primer sequence to validate gene expression of predicted candidate-secreted effector proteins.

Primer name	Sequence (5´-3´)	PCR product size	Melting temperature	*Colletotrichum* gene homology. NCBI Accession
CAC1-F	GCGAGCATAGGTGAAACGTT	206 pb	60 °C	*Colletotrichum legendarium* CAC1 gene for adenylate cyclase, complete cds. Accession AB127957.1
CAC1-R	ATGTTGTTCCTCCGCACGTC			
ClrRNA2	CCTGTTCGAGCGTCATTTCA<br>CCGGTGCGAGGTGGTATG	132 pb	60 °C	*Colletotrichum lindemuthianum* culture CBS:144.31 strain CBS 144.31 internal transcribed spacer 1, partial sequence; 5.8S ribosomal RNA gene and internal transcribed spacer 2, complete sequence; and large subunit ribosomal RNA gene, partial sequence. Accession: MH855161.1
ITS-1	TCCGTAGGTGAACCTGCGG	587 pb	60 °CC	*Colletotrichum sublineola* isolate Cs02 small subunit ribosomal RNA gene Accession: OQ727072.1
ITS-4	TCCTCCGCTTATTGATATGC			
