# Supplementary material for: Morphological and transcriptional analysis of Colletotrichum lindemuthianum race 7 during early stages of infection in common bean
Source: Genet Mol Biol. 2024 Apr 8;47(1):e20220263. doi: 10.1590/1678-4685-GMB-2022-0263 (PMC11003654; doi:10.1590/1678-4685-GMB-2022-0263)
Supplement: Table S2 - [file 1415-4757-GMB-47-01-e20220263-s2.pdf]

Genetics and Molecular Biology

https://doi.org/10.1590/1678-4685-GMB-2022-0263

Supplementary Material to Morphological and transcriptional analysis of *Colletotrichum lindemuthianum* race 7 during early stages of infection in common bean

Table S2 - Quantity and quality of reads from 24, 48, and 72 hours after inoculation. Control (C) and inoculated (I) samples are presented.

ID sample	Total reads	Raw data (Gb)	Q20 (%)	Q30 (%)	GC (%)
24-C-1	51,193,640	7.7	97.72	93.55	45.18
24-I-12	40,933,272	6.1	97.29	92.73	44.88
24-I-22	44,852,728	6.7	97.33	92.80	44.76
48-C-2	69,040,724	10.4	97.68	93.47	45.53
48-I-11	53,271,146	8.0	97.99	93.99	46.82
48-I-22	39,980,354	6.0	97.92	93.85	45.09
72-C-2	45,779,602	6.9	97.61	93.37	45.14
72-I-11	46,272,028	6.9	97.69	93.44	45.16
72-I-22	45,735,440	6.9	97.77	93.57	45.30
