# Supplementary material for: Morphological and transcriptional analysis of Colletotrichum lindemuthianum race 7 during early stages of infection in common bean
Source: Genet Mol Biol. 2024 Apr 8;47(1):e20220263. doi: 10.1590/1678-4685-GMB-2022-0263 (PMC11003654; doi:10.1590/1678-4685-GMB-2022-0263)
Supplement: Table S3 - [file 1415-4757-GMB-47-01-e20220263-s3.pdf]

Genetics and Molecular Biology

https://doi.org/10.1590/1678-4685-GMB-2022-0263

Supplementary Material to Morphological and transcriptional analysis of *Colletotrichum lindemuthianum* race 7 during early stages of infection in common bean

Table S3 - Number of total reads concatenated for *Colletotrichum lindemuthianum* race 7, obtained after preprocessing and filtering for fungi kingdom.

Sample	# Total concatenated reads	# Total Preprocessing Reads (*Trimming*)	# Total reads belonging to Fungi	Filtering (%)
24-F	42,893,000	37,224,018	1,797,959	4.83
24-R	42,893,000	37,224,018	1,797,959	4.83
48-F	46,625,750	42,600,687	2,277,685	5.35
48-R	46,625,750	42,600,687	2,277,685	5.35
72-F	46,003,734	41,166,966	1,660,766	4.03
72-R	46,003,734	41,166,966	1,660,766	4.03
