# Supplementary material for: Morphological and transcriptional analysis of Colletotrichum lindemuthianum race 7 during early stages of infection in common bean
Source: Genet Mol Biol. 2024 Apr 8;47(1):e20220263. doi: 10.1590/1678-4685-GMB-2022-0263 (PMC11003654; doi:10.1590/1678-4685-GMB-2022-0263)
Supplement: Figure S2 - [file 1415-4757-GMB-47-01-e20220263-s5.pdf]

Genetics and Molecular Biology

https://doi.org/10.1590/1678-4685-GMB-2022-0263

Supplementary Material to Morphological and transcriptional analysis of *Colletotrichum lindemuthianum* race 7 during early stages of infection in common bean

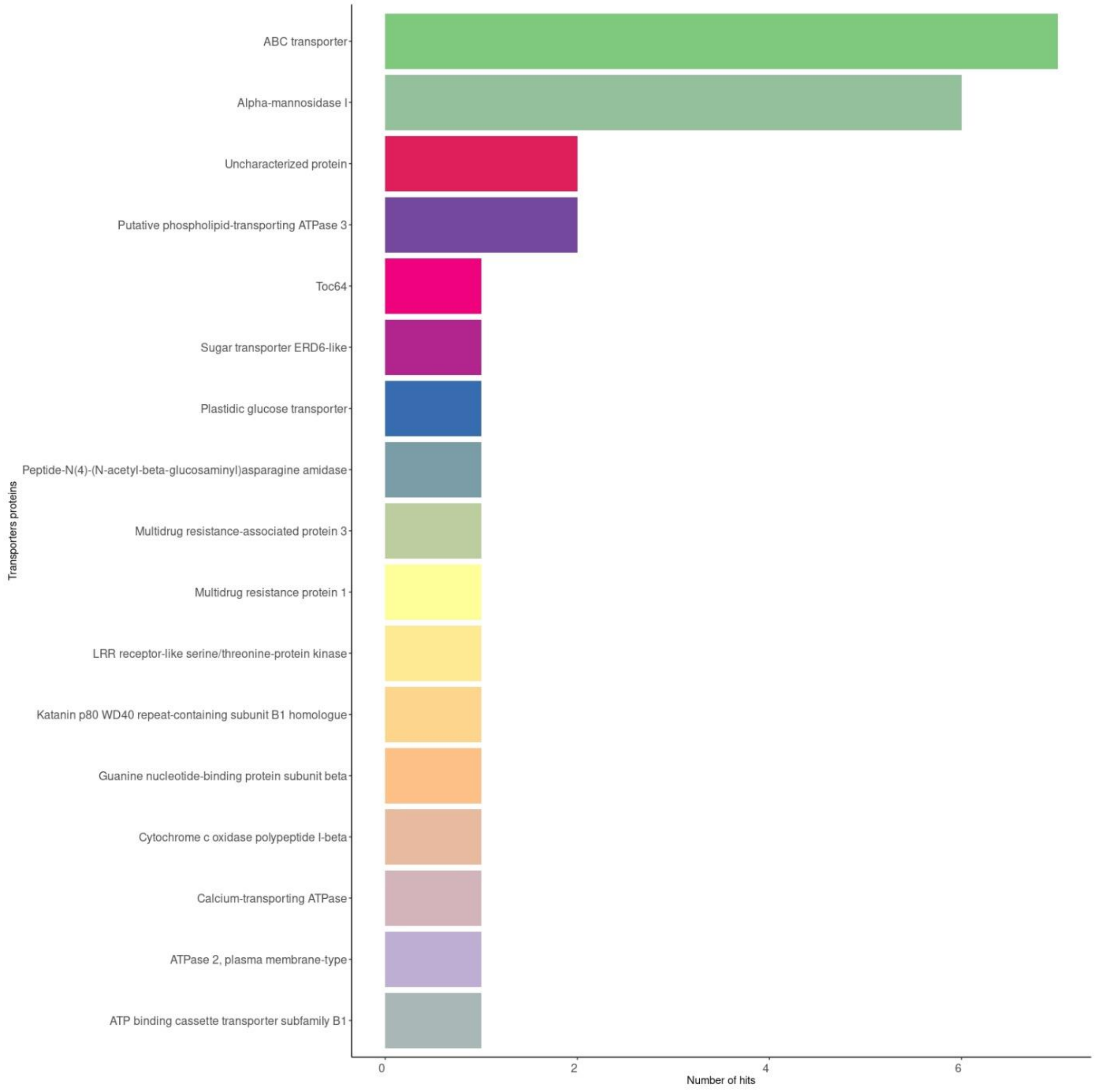

Figure S2 - Transport proteins of *Colletotrichum lindemuthianum* race 7 identified in the Transporter Classification Database.
